# A Framework Including Recombination for Analyzing the Dynamics of Within-Host HIV Genetic Diversity

**DOI:** 10.1371/journal.pone.0087655

**Published:** 2014-02-07

**Authors:** Ori Sargsyan

**Affiliations:** Independent Researcher, Los Alamos, New Mexico, United States of America; British Columbia Centre for Excellence in HIV/AIDS, Canada

## Abstract

This paper presents a novel population genetic model and a computationally and statistically tractable framework for analyzing within-host HIV diversity based on serial samples of HIV DNA sequences. This model considers within-host HIV evolution during the chronic phase of infection and assumes that the HIV population is homogeneous at the beginning, corresponding to the time of seroconversion, and evolves according to the Wright-Fisher reproduction model with recombination and variable mutation rate across nucleotide sites. In addition, the population size and generation time vary over time as piecewise constant functions of time. Under this model I approximate the genealogical and mutational processes for serial samples of DNA sequences by a continuous coalescent-recombination process and an inhomogeneous Poisson process, respectively. Based on these derivations, an efficient algorithm is described for generating polymorphisms in serial samples of DNA sequences under the model including various substitution models. Extensions of the algorithm are also described for other demographic scenarios that can be more suitable for analyzing the dynamics of genetic diversity of other pathogens *in vitro* and *in vivo*. For the case of the infinite-sites model, I derive analytical formulas for the expected number of polymorphic sites in sample of DNA sequences, and apply the developed simulation and analytical methods to explore the fit of the model to HIV genetic diversity based on serial samples of HIV DNA sequences from 9 HIV-infected individuals. The results particularly show that the estimates of the ratio of recombination rate over mutation rate can vary over time between very high and low values, which can be considered as a consequence of the impact of selection forces.

## Introduction

Recombination has an important role in shaping the dynamics of within-host HIV genetic diversity, particularly making the virus capable of escaping the pressures of antiviral drugs and immune system [1], [2]. Therefore, it is of great interest to model this process at the within-host HIV population genomic level. In contrast to the existing methods in the literature, this paper develops a novel population genetic model including recombination and a computationally and statistically tractable framework for this model to explore the dynamics of within-host HIV genetic diversity based on serial samples of HIV DNA sequences.

Early studies [3]–[8] analyzed serial samples of HIV DNA sequences from HIV-1-infected individuals by developing frameworks based on statistically and computationally tractable models, which, however, neglect the impact of recombination or show less mimicking power for the dynamics of within-host HIV genetic diversity. Such an example is the standard coalescent model [9]–[13] that represents a continuous approximation for genealogical and mutational processes for samples of DNA sequences under the Wright-Fisher model with constant population size but without recombination. Another example is the ancestral recombination graph [14], [15] that extends the standard coalescent by including recombination. Shriner et al [16] used various computational tools [17]–[19] based on this model to infer within-host HIV recombination rate. Although both models are attractive because of computational and statistical tractability, the expected dynamics of genetic diversity in serial samples of DNA sequences under these models are inconsistent with observed dynamics of within-host HIV genetic diversity.

**Figure 1:**
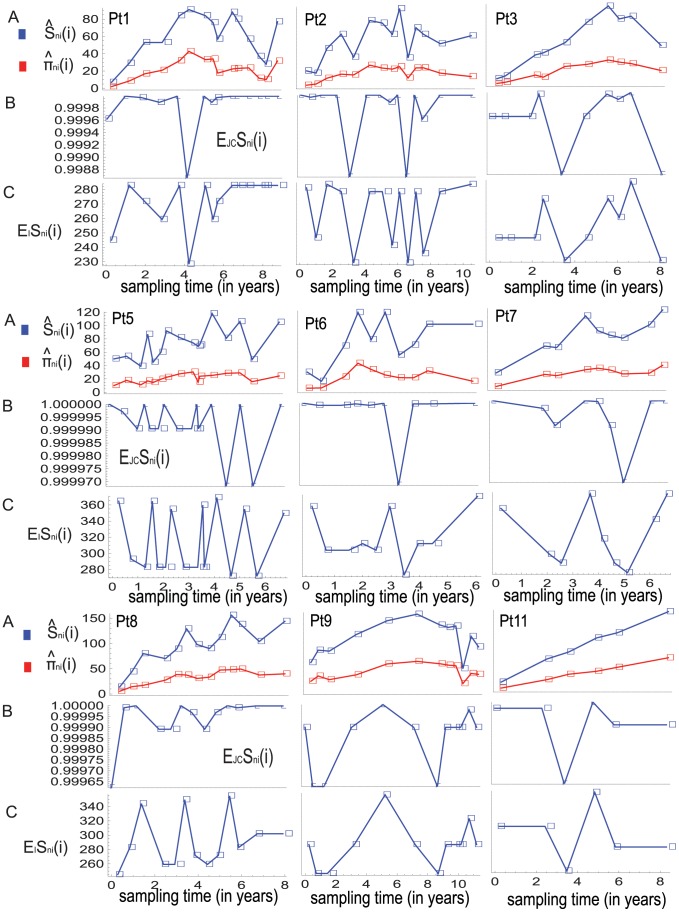
The observed and expected numbers of polymorphic sites and average pairwise differences in serial samples. The horizontal axis of each panel indicates sampling time since seroconversion. (A) The observed numbers of polymorphic sites, 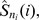 and average numbers of pairwise differences, 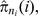 in serial samples are plotted with respect to the sampling times; the data points determined by the two statistics are connected by blue and red lines, respectively. (B) and (C) show the expected numbers of polymorphic sites in serial samples under the Wright-Fisher model with constant population size combined with the finite-sites Jukes-Cantor model, as well as with the infinite-sites model, respectively. Under these substitution models, the expected values of this statistic for sample size 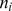 at time 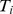 are denoted by 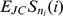 and 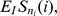 respectively. The expected average numbers of pairwise differences for the serial samples in each individual's case are not shown since they are the same for the samples.

**Figure 2:**
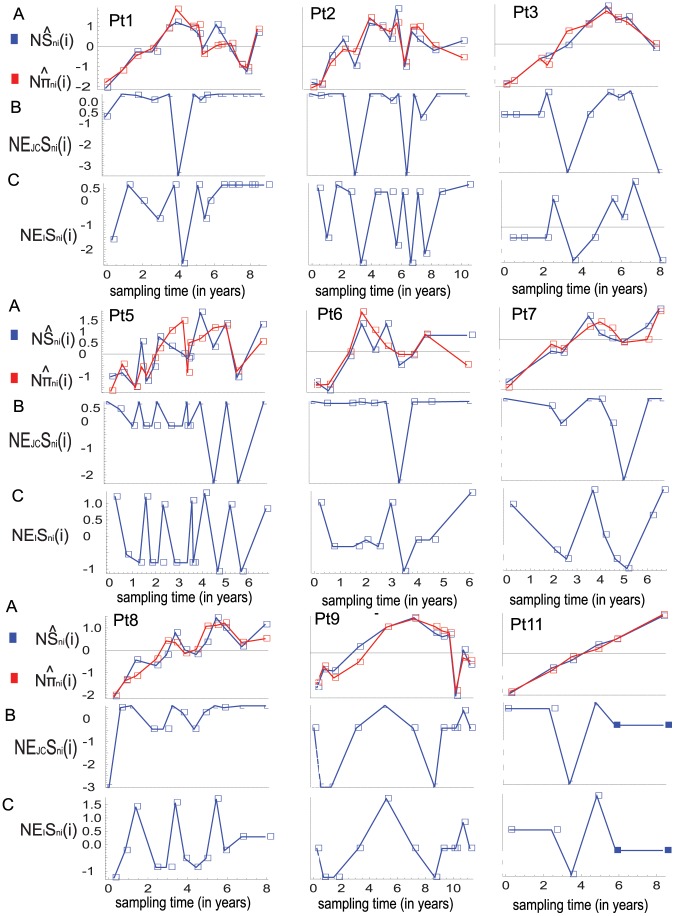
Normalized observed and expected values of the two summary statistics. (A) The dynamics of the observed values of the two statistics in serial samples (Figure 1) are normalized based on transformation (1) and denoted by 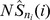 and 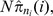 respectively. (B) and (C) show the normalized values of the expected numbers (Figure 1) of polymorphic sites in serial samples under the finite-site Jukes-Cantor model and the infinite-sites model denoted by 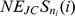 and 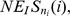 respectively. The normalized values of the expected average numbers of pairwise differences in serial samples are equal to 0 and are not plotted.

Under these models the expected numbers of average pairwise differences in serial samples stay the same [20] and independent on recombination rate [15]. This is a consequence of a more general fact that samples of DNA sequences at different time points under these models are not affected by temporal factor because the distributions of the polymorphisms in equal-size temporal samples are the same due to the same genealogical and mutational processes. In contrast to these expectations, Shankarappa et al [21] observed that the divergences and average numbers of pairwise differences in serial samples of HIV DNA sequences from 9 HIV-1-infected individuals increased linearly for several years after seroconversion but declined or stabilized late in the infection (see Figures 1 and 2 by [21]).

From the same data sets, I also observe linear relationships between the dynamics of the numbers of polymorphic sites and the numbers of average of pairwise differences in each individual's case (Figure 1). To make the linear relationships more obvious, I use the following normalization because two data sets are linearly related if and only if their normalizations are the same. Let 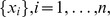 be the values of one of the statistics in serial samples. If 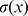 is not equal to 0, where 
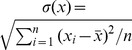
 and 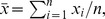 then the normalized values are
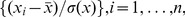
(1)otherwise they are set to be 0. Figure 2 shows the dynamics of the normalized values of the two statistics from the observed samples.

To illustrate non-linear relationship between the expected dynamics of the two statistics for the observed sample sizes under the Wright-Fisher model with constant population size, I compute the expected numbers of polymorphic sites under the finite-sites Jukes-Cantor model as well as under the infinite-sites model by using the formulas of Tajima [22] and Watterson [23], respectively. Figure 1 shows the expected numbers of polymorphic sites for the observed sample sizes under both mutational models. The normalized values of the expected numbers of polymorphic sites are in Figure 2, which makes obvious the non-linear relationship between the expected dynamics of the two statistics due to the differences in the sample sizes.

While the standard coalescent as well as the ancestral recombination graph might not be directly applicable for analyzing the dynamics of HIV genetic diversity within a host, the concepts and features of these models had and have great impact on extending coalescent theory for other evolutionary settings. Both models were derived as continuous limits of discreet genealogical and mutational processes under the Wright-Fisher models with constant population size and with and without recombination by applying the time scaling concept that is measuring time proportional to a very large population size. This concept was also applied for other forward in time Wright-Fisher models with variable population size, selection, or migration but without recombination (see e.g. [15], [24], [25]) to derive continuous coalescent models. An attractive feature of continuous approximations is that the models are computationally and statistically tractable for analyzing samples of DNA sequences by efficiently generating samples of DNA sequences and combining or contrasting the generated data sets with observed data.

Later studies [26]–[30] combined the variable-population-size coalescent model [31] with phylogenetic methods to derive frameworks for analyzing serial samples of DNA sequences. However, these methods ignore recombination, and that can be a significant limitation, particularly, for analyzing serial samples of HIV DNA sequences because HIV genome is strongly affected by recombination [32]. Furthermore, Schierup and Hein [33] showed that the standard phylogenetic methods ignoring recombination can result in misleading inference. Other tools for analyzing serial samples under the variable population size model were also developed by Anderson et al [34] and Jakobsson [35] by using the discrete approximation method of Excoffier et al [36]. In this method the genealogy of sequences are constructed by tracing their lineages generation-by-generation back in time, and this gives an advantage of easily adapting this approach for other evolutionary scenarios including recombination. However, this method is computationally less efficient in comparison to the continuous approximation based methods in which the genealogies of sequences are constructed by tracing consecutive coalescent and recombination events back in time.

To overcome the limitations of the previous models and methods mentioned above, I first describe a forward in time population genetic model to represent HIV evolution in HIV-infected individuals in the chronic phase of infection. The population in the model is considered to be homogeneous at the beginning, representing the time of HIV seroconversion, and to evolve according to the Wright-Fisher reproduction model with recombination by allowing the population size and generation time to vary over time. To make this model computationally and statistically tractable for analyzing serial samples of DNA sequences, I apply the time scaling approach at multiple time intervals (instead of a single time interval as in previous methods) to describe a continuous coalescent-recombination process for tracing the lineages of the samples back in time and superimposing mutational events on the lineages according to an inhomogeneous Poisson process. Based on these processes I describe computationally efficient algorithm for generating polymorphisms in serial samples of DNA sequences drawn randomly under this population genetic model. Further extensions of the algorithm are also described for population genetic models that can be more suitable for analyzing the dynamics of genetic diversity of other pathogen populations in vivo and in vitro.

Within this framework I consider two substitution models: a finite-sites model with variable mutation rate across nucleotide sites and the infinite-sites model. For the infinite-sites case, I derive analytical formulas for the expected number of polymorphic sites in samples of DNA sequences. For this quantity, Tajima [37] also derived an analytical expression by using a different approach. Thus, the developed simulation and analytical methods I apply to serial samples of HIV DNA sequences from 9 HIV-infected individuals [21] to explore the fit (the mimicking power) of the model to the data sets and to identify and quantify the signatures of recombination and selection on the dynamics of within-host HIV genetic diversity. Within this analysis I particularly explore the contrasting estimates for within-host HIV recombination rate by previous studies [16], [38], [39].

## Methods

### The population genetic model

To model within-host HIV evolution, I take into account the following observations: (1) HIV population within HIV-infected individuals usually collapses at seroconversion (the onset of the antiviral immune response) after several weeks of infection and recovers quickly as a homogeneous population [40]–[42]. (2) The viral load and CD4+ cell counts within HIV-infected individuals change over time, and I take this as an intuitive base for considering variability for within-host HIV generation time and population size. Thus, I consider a population model that is homogeneous at the beginning (representing the time of seroconversion) and evolves according to the neutral Wright-Fisher reproduction model with recombination, in which the population size and generation time vary over time but stay constant between consecutive sampling time points.

In this model DNA sequences are represented as a combination of 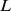 consecutive loci, each of which consists 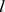 nucleotides. Recombination events along the lineages per sequence per generation occur with rate 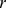 and recombinations (crossovers) between two sequences are allowed only at the breakpoints between the consecutive loci. Mutation events per sequence per generation occur with rate 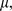 and the substitutions at the nucleotide sites occur according to a finite-sites model. Although various finite-sites substitution models [43]–[48] can be incorporated within this model, I only consider the infinite-sites model and a finite-sites Jukes-Cantor model with variable mutation rate across nucleotide sites. Note that the infinite-sites model is a limiting case of the finite-sites model by considering the locus length 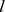 to be very large.

I design the finite-sites model in a such way that it represents the heterogeneity of substitution rate across nucleotide sites and infer some of the parameters in this model based on serial samples of HIV-1 DNA sequences from envelop gene region [21]. In this model the sequences are combinations of two regions 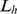 and 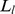 with high and low mutation rates, respectively. To have this contrast, I assume that region 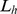 has less nucleotides than region 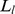 and mutations occur uniformly within the regions with the same rate 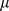 per generation per region. In this scenario nucleotide changes at mutation events occur according to the Jukes-Cantor model [43].

The population model for 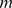 serial samples is parameterized as follows. Let 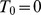 be the time of seroconversion, and the sampling time points since seroconversion are labeled as 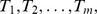 in chronological order, measured in years, days, or hours. Let 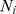 and 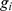 be respectively the population size and generation time for time interval 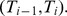 The collection of these parameters are represented as 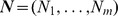 and 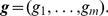 The sample size at time 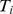 is denoted by 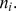


## Results

Variation in a sample of DNA sequences under the above population model can be described by combining the genealogical history of the sequences with mutations on the lineages of the sequences. The genealogical history traces the ancestral lineages of the sequences back in time before time 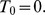 I approximate this process by a continuous coalescent-recombination process described as follows. I consider 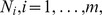 to be large and measure time in time interval 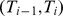 by 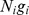 time units and approximate the genealogical history of the sample in this interval by the ancestral recombination graph [14], [15] with scaled recombination rate 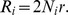 This ancestral graph is the same as the one derived under the Wright-Fisher model with constant population size 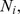 generation time 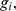 and recombination rate 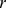 per sequence per generation. Note that instead of using a single time scaling as in previous studies, I scale time in multiple time intervals, which gives an advantage to derive a continuous coalescent-recombination process for the above described population genetic model.

For each interval 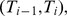 the tracing procedure maps to a continuous-time Markov chain for which time varies between 0 and 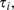 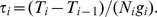 The chain is described by the transition time 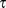 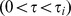 and the number of lineages at time 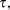 denoted by 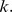 Initially, the values of 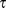 and 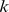 are set respectively equal to 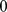 and 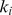, where 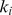 is the total number of sequences in two sets: one set includes the sampled 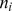 sequences at time point 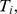 the other set represents the sequences at time 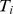 that are linked to the lineages traced between time points 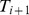 and 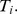 A possibility of overlap between the two sets are ignored since 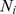 is considered to be large.

For this Markov chain the transition from the state 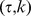 is described as follows: First, a random number 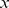 is generated from an exponential distribution with parameter 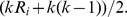 If 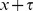 is greater than 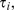 the 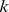 lineages are traced “straight” before time point 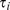; the value of 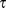 is updated by the value of 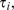 and the procedure for this time interval stops. Otherwise, the value of 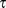 is updated by the value of 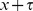 and the lineages are traced before time 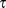. The set of 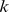 sequences linked to the lineages at time 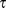 are modified by a coalescent event with probability 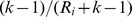 or a recombination event with probability 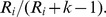 In the case of coalescent event, two lineages are randomly chosen out of the 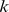 lineages and merged into a single lineage. The set of the sequences at this time point is updated by replacing the two sequences of the merging lineages with a single sequence and decreasing the value of 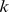 by 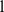. Otherwise (in the case of recombination) creating two new sequences by randomly choosing one of the 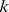 sequences and one of the 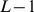 breakpoints and copying left and right segments of that sequence with respect to that breakpoint into two sequences. The chosen sequence is discarded from the set of 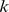 sequences. If any of the two new sequences does not share an ancestral segment with the sequences sampled at time 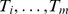, that sequence is also discarded and the other one added to the set of 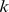 sequences. Otherwise, the two new sequences are added to the set of the 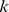 sequences. In the latter case the value of 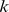 is increased by 1. Recursively repeating the steps and updating values of 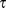 and 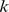, the procedure continuously traces the lineages before time 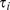.

The following algorithm uses the tracing procedure recursively to describe a bottom up process for generating genealogical history of serial samples and a top down process for adding mutation events on the genealogy. The algorithm can be used to generate variation in serial samples under the population model described above.

*Algorithm* 1

1. Set the values of 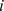, 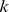, and 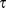 equal to 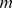, 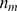, 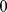, respectively.
2. Apply the above procedure for tracing the lineages of the 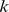 sequences in the time interval 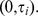

3. Update the values of 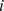, 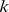, and 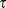 by the values of 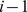, 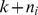, 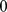, respectively.
4. As the value of 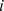 is greater than 0, go to Step 2. Otherwise the genealogical history of the samples is generated. Update the value of 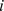 by 1
5. Add mutation events independently on different branches of the genealogical history for time interval 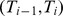 according to a Poisson process with rate equal to 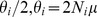. At each mutation event, introduce mutational changes in the sequences according to the finite-sites model.
6. Increase the value of 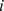 by 1. Stop if the value of 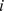 is greater than 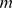, otherwise go to Step 5.

For the case of a non-recombining locus (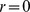), the algorithm can be extended to include deterministic fluctuations in population size. Let 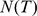 be a function determining the population size at time 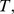 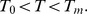 Assuming that it is a continuous function in each of the intervals 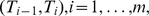 and its limit at 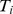 from left is denoted by 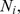 which is 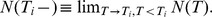 Based on this notations and the transformation functions 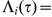 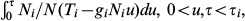 defined by [49], I derive the following algorithm by modifying Algorithm 1.

*Algorithm* 2

1. Set the values of 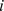, 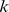, and 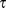 to be equal to 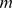, 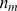, 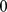, respectively.
2. Apply the above procedure for tracing the lineages of the 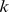 sequences for time interval 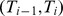 by generating the Markov chain for time interval 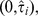 where 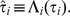

3. Update the values of 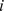, 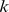, and 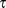 by the values of 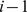, 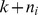, 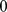, respectively.
4. If the value of 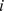 is greater than 0 go to Step 2. Otherwise update the value of 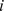 by 1.
5. Transform the branch lengths of the generated genealogical history for time interval 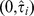 by applying the inverse of the function 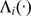 to the coalescence waiting times in that part of the genealogy. (For example, if the part of the generated genealogical history for time interval 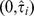 starts with 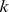 lineages and 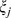 is the waiting time for the number of the lineages to decline to 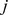 first time, then the corresponding coalescence time 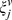 in the variable population size case is determined by the equation 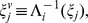 where 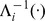 is the inverse of 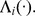

6. Add mutation events independently on different branches of the part of the transformed genealogy according to a Poisson process with rate equal to 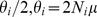. At each mutation event, introduce mutational changes in the sequences according to the finite-sites model.
7. Increase the value of 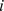 by 1. Stop the process if 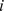 is greater than 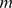, otherwise go Step 5.

In the case of a non-recombining locus the process described in Algorithm 1 for constructing the genealogical history of 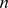 sequences sampled at time 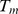 can be simplified: instead of considering 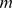 tracing procedures for time intervals 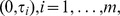 the genealogy of the sample can be constructed by a single tracing procedure for time interval 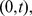 where 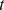 is equal to 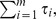 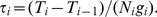 This simplification is a result of the fact that the waiting times to the coalescent events in the tracing process are exponential random variables and satisfy the memoryless property.

One implication of this result is that it allows to derive an analytical formula for the expected number of polymorphic sites in a sample of DNA sequences drawn randomly from the piecewise constant population size model combined with the infinite-sites model. Another analytical expression for the same quantity was also derived by Tajima [37] using recursion formulas for expected numbers of polymorphic sites.

**Lemma 1** *Let 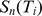 be the number of polymorphic sites in a sample of 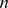 DNA sequences drawn from the above population model at time 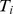. The mean of 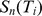 can be computed by using the formula *

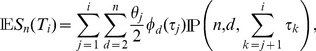
(2)where 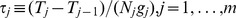 *. The functional* 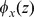 *is the expected total branch length of the genealogy constructed by tracing* 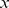 *sequences according to the above procedure starting at 0 and ending at the latest time point before* 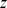 *and the most recent common ancestor;* 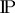 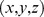 *is the probability that* 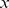 *sequences traced according to the described procedure have* 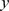 *ancestors at time* 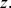 *Analytical expressions for* 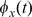 *and* 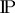 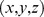 *were derived by* [37], [50]–[52] *and* [49] *, respectively.*

The proof of the lemma is in Appendix S1.

Note that Tajima [37] derived the formula for 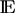 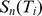 including the non-homogeneous population case at time 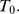 To include that case in the formula (2), the expression 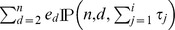 should be added to the right of equation (2). The quantity 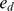 represents the expected number of polymorphic sites in a sample of 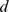 sequences drawn from the population at time 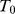. Particularly, if the population before time 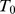 is modeled according to the Wright-Fisher model with constant population size, 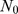, then 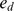 is equal to 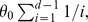 according to Watterson's formula [23], in which 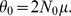


The formula (2) also holds for the case of 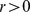 because the genealogies at the non-recombining parts of the sequences are identically distributed. Note that this formula also allows to compute the expected average number of pairwise differences in samples because that quantity is equal to the expected number of polymorphic sites in randomly chosen two sequences. Note that this formula can also be applied for computing the expected average number of pairwise differences between two sequences sampled at two different time points. Let 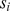 and 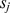 be DNA sequences drawn from the above population model at times 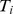 and 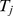, 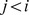, respectively; and 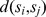 is the number of sites at which the sequences 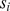 and 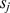 differ. The expected value of 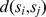 can be computed by using the formula
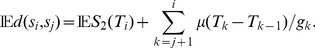
(3)


### Application of the framework

I apply the models and methods described in the previous section for exploring within-host HIV evolution based on serial samples of HIV DNA sequences from 9 HIV-1-infected individuals studied by [21]. As in that study, I also use the same identifiers for the 9 individuals: Pt1, Pt2, Pt3, Pt5, Pt6, Pt7, Pt8, Pt9, Pt11. The serial samples were based on blood specimens provided by the individuals at their semiannual visits since seroconversion. The sequences are from C2-V5 region of the HIV-1 envelop gene and are 764 bases long including nucleotides and insertion/deletions based on the alignments of all the sequences.

First, I fit the population genetic model to data sets under the finite-sites model, in which the values of 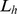 and 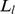 are estimated from the alignments of all the sequences in the serial samples for each individual's case by classifying the nucleotide sites into three groups. Group 1 includes nucleotide sites that are conserved; group 2 represents sites at which only two nucleotides are present; the rest of the sites are in group 3: sites at which more than 2 nucleotides are present. Table 1 shows the sizes of the groups 2 and 3 that determine the values of 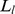 and 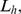 respectively.

**Table 1:** The sizes of the groups of classified nucleotide sites based on the alignments of DNA sequences in serial samples for each individual's case.

individual	group 2^a^	group 3^b^
Pt1	240	70
Pt2	307	155
Pt3	181	59
Pt5	275	104
Pt6	215	49
Pt7	204	88
Pt8	287	88
Pt9	257	102
Pt11	209	46

a Group 2 includes polymorphic sites at which only two nucleotides are present.

b Group 3 includes polymorphic sites at which more than two nucleotides are present.

**Figure 3:**
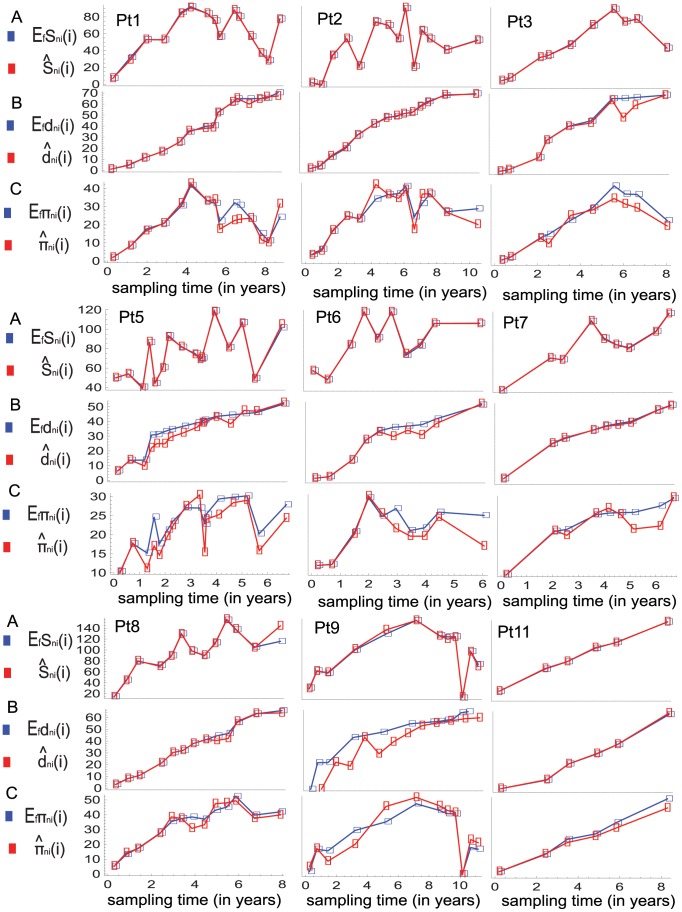
The fit of the model to the data in the finite-sites model case. In this case the population genetic model is fitted to the data by matching the observed values of the numbers of polymorphic sites, 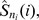 and divergences, 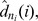 in the serial samples to their expected values, denoted by 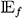 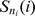 and 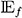 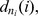 respectively. (A) shows the observed and fitted (expected) values of the numbers of polymorphic sites in serial samples. The observed and expected data points are connected by red and blue lines, respectively. (B) shows the observed and fitted (expected) values of the divergences in serial samples. Based on this fitting the vectors 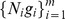 and 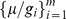 are estimated, and for the fitted model the predicted (expected) values of the average numbers of pairwise differences in serial samples are computed. (C) shows the observed and predicted values of this statistic in the serial samples, and the statistics are denoted by 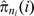 and 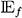 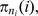 respectively.

I implemented Algorithm 1 for this mutation model into a computer program (in C programming language) for generating the polymorphisms in serial samples and applying the Monte Carlo approach to estimate the expected values of summary statistics for the observed sample sizes. To fit the model to the data sets for each individual's case, I consider two summary statistics: the numbers of polymorphic sites and divergences in the serial samples. Divergence in a sample of sequences is defined as the average of the numbers of differences between the founder sequence and the sequences in the sample. The founder sequence is the sequence of the homogeneous population at the beginning; and for the observed samples, the founder sequence is inferred from the alignment of the sequences in the first sample taken (at time 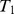) after seroconversion. The most frequent nucleotide at each nucleotide site in the alignment of the sequences in that sample is defined as the nucleotide of the founder sequence. To estimate the parameters 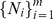 and 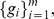 I recursively match the observed values of the two statistics to their closest expected values. Thus, I first estimate the values of 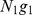 and 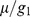 and then recursively estimate the other elements of the vectors 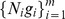 and 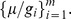 The parameter vectors 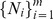 and 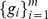 are estimated up to a constant factor 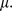 Figure 3 shows the fitted dynamics of the expected values of the two statistics to their observed values in the serial samples.

**Figure 4:**
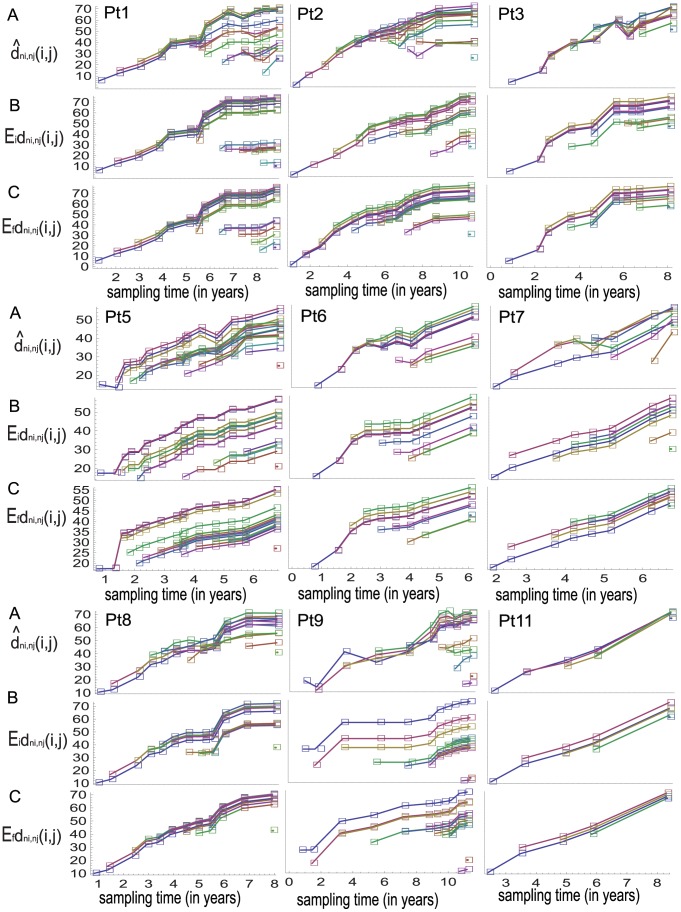
Observed and expected average numbers of pairwise differences between sequences at different sampling time points. Average number of pairwise difference between sequences in samples taken at times 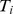 and 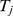 are denoted by 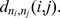 The observed and expected values of this statistic under the infinite-sites model and the finite-sites model are denoted by 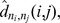 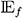 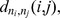 and 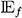 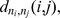 respectively. (A) shows the observed values of 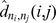 in the serial samples for each individual's case. (B) and (C) show the predicted (expected) values of 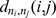 in the serial samples for each individual's case computed receptively under the fitted models for the cases of the infinite-sites and finite-sites models.

To assess the overfitting of the estimated model to the data, I consider two additional statistics: the average numbers of pairwise differences between and within the serial samples. Figures 3 and 4 show the observed and expected dynamics of the two statistics, in which the expected (predicted) values of the two statistics are estimated under the fitted model. The statistics are used as controls, and the overall fit of the estimated model in each individual's case is quantified by the overall-fit score defined as follows. The observed and expected dynamics of the four statistics (computed under the fitted model) in the serial samples are represented as vectors of numbers and the overall-fit score is defined as the sum of the Euclidean distances between the observed and expected vectors. Thus, the overall-fit scores carry the tread-off between fit and prediction for the estimated models in the content of the four statistics.

**Figure 5:**
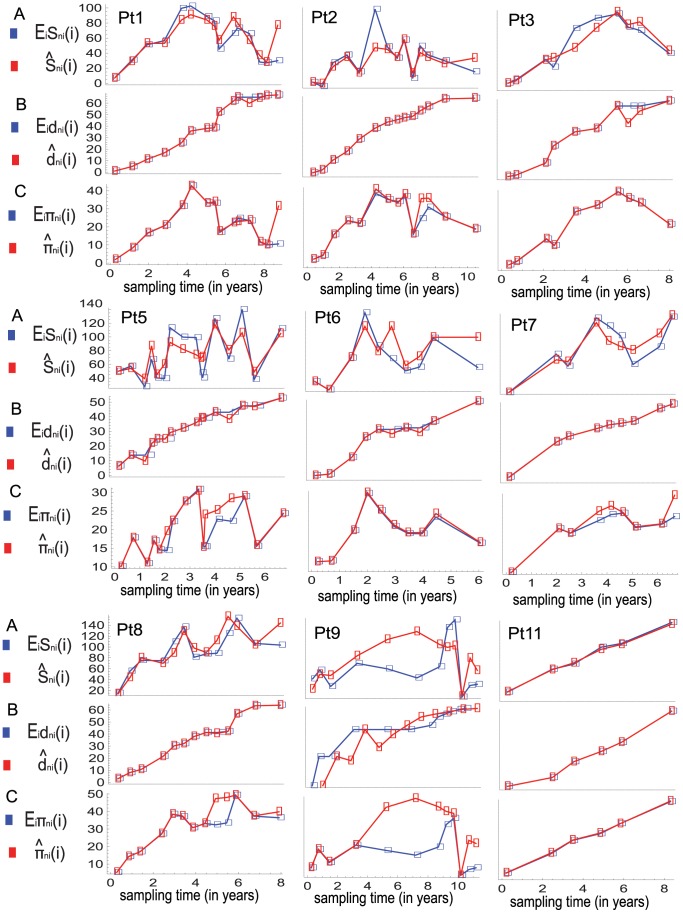
The fit of the model to the data in the infinite-sites model case. In this case the population genetic model is fitted to the data by matching the observed values of the numbers of polymorphic sites, 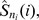 and divergences, 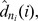 in the serial samples to their expected values, denoted by 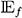 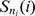 and 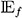 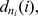 respectively. (A) shows the observed and fitted (expected) values of the numbers of polymorphic sites in serial samples. The observed and expected data points are connected by red and blue lines, respectively. (B) shows the observed and fitted (expected) values of the divergences in serial samples. Based on this fitting the vectors 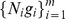 and 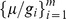 are estimated, and for the fitted model the predicted (expected) values of the average numbers of pairwise differences in serial samples are computed. (C) shows the observed and predicted values of this statistic in the serial samples and are denoted by 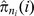 and 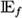 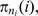 respectively.

I also fit the model to the data sets under the infinite-sites model and compare the mimicking powers of the two models with respect to the data sets by using graphical assessment as well as using the overall-fit scores. The estimation procedure is the same as in the previous case except that the analytical formulas (2) and (3) are used for computing the expected values of the four statistics. In this case the estimated expected values of the four statistics do not show strong qualitative discrepancy with their observed values except in the case of individual Pt9 (see Figure 5 and 4). Table 2 shows the overall-fit scores of the two models for each individual's case. Thus, the estimated finite-sites model shows better qualitative and quantitative mimicking power in each individual’s case.

**Table 2:** The overall-fit scores of the population genetic models to the data sets from each individual.

individual	score 1^a^	score 2^b^	score 3^c^
Pt1	119	184	17063
Pt2	89	246	15603
Pt3	70	102	1529
Pt5	90	143	40184
Pt6	53	83	1850
Pt7	37	83	4417
Pt8	81	127	6221
Pt9	145	383	154575
Pt11	23	20	28160

a In the fitting process the observed numbers of polymorphic sites and divergences in the serial samples are matched to their expected values under the population genetic model in the finite-sites model setting.

b The fitting process is the same as in case of score 1 except that the infinite-sites model is considered.

c The overall-fit scores are computed in the content of the four statistics from a model that is estimated by matching the observed numbers of polymorphic sites and average numbers of pairwise differences in the serial samples to their expected values under the model in the infinite-sites setting.

The contrast between the mimicking powers of the two mutation models in the content of the four statistics is more obvious when I fit the population genetic model to the data sets under the infinite-sites model by matching the observed values of the numbers of polymorphic sites and average numbers of pairwise differences in the serial samples to their expected values, and I use the other two statistics as controls. The overall-fit scores in this case are also in Table 2. Note that in this case the expected (predicted) values of divergence over time increase much faster and have bigger values than the observed values (details not shown) which are less than 100, but the sequences are about 764 bases long. Intuitively, such discrepancy can be controlled by considering a finite-sites model in which sequence length is much smaller than 764 bases, and this intuition was one of the reasons for considering the finite-sites model descried in this paper.

### The signature of recombination on the dynamics of within-host HIV genetic diversity

I use the estimated models for the case of the finite-sites model (described in the previous section) to explore the signature of recombination on the dynamics of HIV genetic diversity. For this purpose I choose two statistics based on the linkage disequilibrium measure 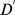 [53], [54] since the expected values of the four statistics described in the previous section are independent on the recombination rate. The two statistics are denoted by 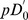 and 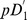 and described as follows. To determine the values of these statistics for a sample of DNA sequences, I first compute 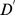 for all pairs of polymorphic sites in the sample by using the formula 14 by [54], which extends the definition of 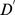 for polymorphic sites with multiple alleles. The statistics 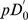 and 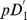 represent the proportion of the computed 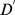 that are equal to 0 and 1, respectively. Intuitively, one can expect 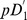 and 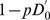 to decrease as 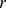 increases since recombination breaks down linkage disequilibrium between nucleotide sites. For the case of two biallelic linked polymorphic sites created by two mutations, 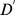 is 0 when the sites are in equilibrium (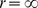) and it is 1 or greater than 0 for completely linked sites (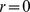).

**Figure 6:**
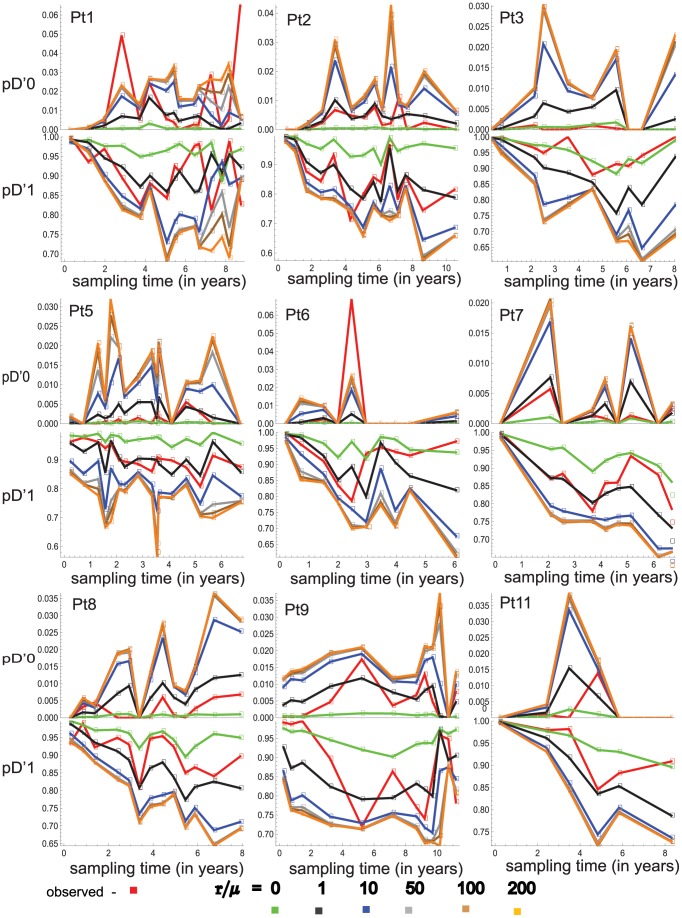
The dynamics of 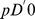 and 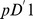 for the serial samples in each individual's case. For the serial samples from each of the individuals, the observed values of 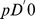 and 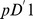 as well as their expected values are plotted with respect to the sampling times. The observed data points are connected by red lines. For each of the values of 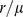 equal to 0, 1, 10, 50, 100, and 200, the expected values of these two statistics are computed by using Monte Carlo approach and Algorithm 1 based on the estimated values of the vectors 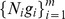 and 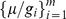 for the finite-sites case.

Using the computer program based on Algorithm 1 and Monte Carlo approach, I estimated the expected values of 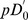 and 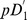 in the serial samples under the estimated models for each individual's case and for each of the values of 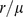 to be equal to 0, 1, 10, 50, 100, 200. The locus length 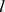 in the simulations is 10 and the numbers of the loci in the regions 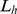 and 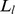 are respectively equal to 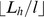 and 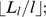 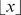 is the greatest integer number that is less than 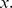 To avoid numerical problems, the values of the two statistics in the simulations are determined by the conditions 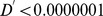 and 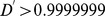 instead of 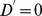 and 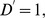 respectively. The expected and observed dynamics of the two statistics in the serial samples for each individual's case are in Figure 6. The results show the following interesting common trends for the dynamics of the two statistics: (1) For most of the individual cases, the observed dynamics of the two statistics fluctuate between the expected dynamics of the two statistics for 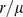 equal to 0 and 1. (2) Some of the observed values of the two statistics are not expected for any of the considered values of 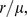 which is particularly obvious for the cases of the individuals Pt1 and Pt6.

**Figure 7:**
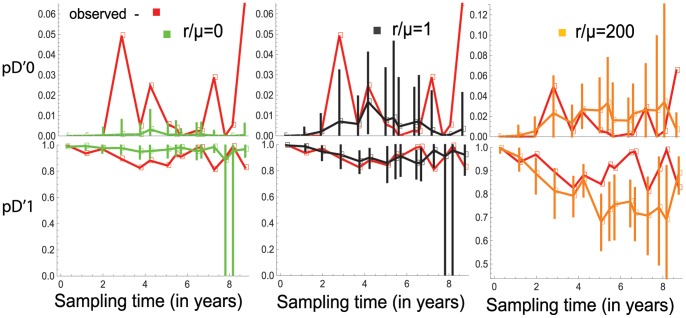
The 95% probability intervals for 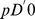 and 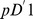 in the case of individual Pt1. The observed values of the statistics 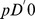 and 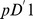 at sampling time points are connected by red lines. For each of the values of 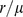 and at each sampling time point the 95% probability interval is inferred by estimating 2.5% and 97.5% quantiles of the statistics under the estimated model in the finite-sites case. The vertical intervals at the sampling time points represent the 95% probability intervals in green, black, and orange when 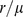 is 0, 1, or 200, respectively. In each case the same colors are respectively used to connect the expected values of the statistics.

In the case of patient Pt1, I explore further and consider three hypotheses for 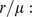 it is equal to 0, 1, or 200. I consider each of the hypotheses as a null hypothesis, and test them for data sets at the sampling time points by estimating 2.5% and 97.5% quantiles of each of the statistics 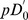 and 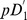 based on the estimated model. Figure 7 shows that each of the hypotheses is rejected at a 5% significance level for some of the data sets at the sampling time points. These trends in the dynamics of the two statistics can be taken as a consequence of selection pressure on the HIV-1 envelop gene region.

## Discussion

### Some modeling issues

The purpose of this study was to develop a computationally and statistically tractable framework, including recombination, for analyzing the dynamics of HIV genetic diversity in HIV-infected individuals. To derive this framework, I first designed a population genetic model that carries some of the features of within-host HIV evolution. Particularly, the model includes recombination, variability in population size and generation time, and heterogeneity of mutation rate across nucleotide sites. In addition, I considered the population size and generation time to vary over time as piecewise constant functions of time; these choices were made in order to derive the framework including recombination and without overwhelming the model with parameters that would be difficult to estimate.

In spite of these choices, the model and framework can be extended for other evolutionary settings. Particularly, the model can be extended to include various distributions for the breakpoints along HIV genome, as well as for mutation rates across nucleotide sites. As another extension of the framework, I described Algorithm 2 that is applicable for serial samples of non-recombining sequences in a more general demographic scenario by allowing the population size to be a piecewise continuous function of time. Such a demographic scenario may be more suitable for exploring evolutionary dynamics of other pathogens at genomic level in vitro and in vivo. Particularly in vitro experiments in which a bacterial population goes through recurrent bottlenecks by growing or declining exponentially over time. However, in this setting the number of the parameters can increase and can be challenging to estimate them. For example, if the population size 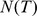 declines or grows exponentially on intervals 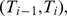 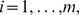 as a function of time 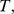 the left and right limits 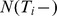 and 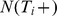 of 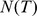 at 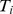 determine this function. Thus, they become parameters of the model, and the total number of parameters can increase at most by 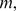 in comparison to the piecewise constant population size case. Exception is the case when 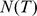 stays constant on interval 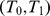 and changes continuously on 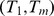 (that is 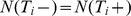).

Another extension of the model is to replace the assumption of homogeneous population at time 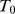 by considering the population to evolve according to the equilibrium Wright-Fisher model before time 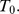 For this case Algorithm 1 should be modified by allowing the lineages of the samples to be traced back in time before the most recent common ancestor. In this setting the genealogical process for a sample of non-recombining DNA sequences is the same as the genealogical process in the standard coalescent because the waiting times between consecutive coalescent events are exponential random variables which have the memoryless property. However, mutation events on the lineages of a such genealogy are added according to an inhomogeneous Poisson process with rate equal to 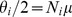 for time interval 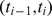, 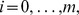 where 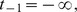 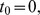 and 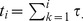 This presentation is consistent with the statement provided by [31], and shows the robustness of the genealogical process in the standard coalescent, which was also observed in other evolutionary settings [15], [24], [25].

Note that the developed framework can be applied to generate HIV transmission chains and HIV epidemics at the genomic level. To be able to accomplish such a task, it is important to have a better understanding of the space of the values of the vectors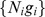 and 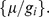 The moment matching approach used in this paper has limited power to assess uncertainties in the estimates of these vectors, this challenge can be overcome with more computational cost by incorporating a rejection method [55] within the developed framework. Such a method might also overcome the fitting limitations of the moment matching method as I observed that the expected dynamics of divergence from the founder sequence show non-decreasing behavior over time but the observed dynamics of this statistic show some fluctuations (see Figures 3 and 5).

### The signatures of recombination and selection on the dynamics of within-host HIV genetic diversity

The application of the framework to the serial samples of HIV DNA sequences from nine HIV infected individuals allowed to explore the fit of the model to the data sets and the impact of the recombination on the dynamics of within-host HIV genetic diversity. Particularly, these results show large variability for inferring the 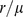 ratio (recombination rate over mutation rate) at different sampling time points (Figures 6). These results are consistent with other studies (see [56] and references therein) that also observed very wide range for estimates of recombination rate in various viruses. Therefore, it is not clear if a particular estimated value of 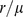 can be taken as a representative value. For example, in the case of individual Pt1 the ratio 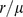 at different sampling time points can be inferred to be 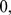 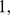 or 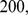 and in the meantime Figure 7 shows that for each of these values there is a time point at which the estimated 95% probability intervals of the statistics 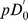 and 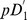 exclude the observed values of these statistics.

The wide spectrum of the inferred values of 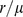 also explains the contrasting estimates of the ratio by other studies. Resent studies [38], [39] used serial samples of HIV DNA sequences and inferred within-host HIV recombination rate to be smaller than the mutation rate. In contrast, Shriner et al [16] inferred within-host HIV recombination rate to be 5.5 fold greater than point mutation rate; they derived the results based on a sample of HIV DNA sequences taken at 2.96 years after seroconversion from individual Pt6 and used estimation tools based on the Wright-Fisher model with constant population size. Thus, the results of this paper suggest that caution should be taken when using a single value estimate of the ratio 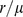 as a representative for quantifying within-host HIV evolution. Note that the same can be applied when using HIV mutation rate per nucleotide per generation because of the variability in mutation rate among nucleotide sites.

Since selection forces have impact on shaping within-host HIV genetic diversity [57], [58], the variability in the estimates of 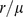 and the extreme observed values of the statistics 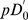 and 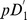 that deviate significantly from their expected values and 95% probability intervals (Figures 6 and 7) can be considered as the signatures of selection forces. However, note that selection and recombination are interconnected processes in shaping within-host HIV genetic diversity, and the presented framework has less power to separate the signatures of the two processes. In the developed population genetic model selection is only included implicitly by considering variable mutation rate across nucleotide sites. To have better understanding the relationship between the two evolutionary processes and their impact on shaping within-host HIV genetic diversity, further modifications are needed in the model.

## Supporting Information

Appendix S1 **The proof of Lemma 1.** This section shows the derivation of the formula (2) by using the developed representation for polymorphisms in samples of DNA sequences under the piecewise constant population size model and the memoryless property of coalescence waiting times in the standard coalescent.
(PDF)

## References

[1] Lemey P, Rambaut A, Pybus OG (2006) HIV evolutionary dynamics within and among hosts. AIDS Rev 8: 125–140. 17078483

[2] Burke DS (1997) Recombination in HIV: an important viral evolutionary strategy. Emerg Infect Dis 3: 253–259. 9284369 10.3201/eid0303.970301 PMC2627633

[3] Rodrigo AG, Shpaer EG, Delwart EL, Iversen AK, Gallo MV, et al (1999) Coa- lescent estimates of HIV-1 generation time in vivo. Proc Natl Acad Sci USA 96: 2187–2191. 10051616 10.1073/pnas.96.5.2187 PMC26758

[4] Fu YX (2001) Estimating mutation rate and generation time from longitudinal samples of DNA sequences. Mol Biol Evol 18: 620–626. 11264414 10.1093/oxfordjournals.molbev.a003842

[5] Seo TK, Thorne JL, Hasegawa M, Kishino H (2002) Estimation of effective popula- tion size of HIV-1 within a host: a pseudomaximum-likelihood approach. Genetics 160: 1283–1293. 11973287 10.1093/genetics/160.4.1283 PMC1462041

[6] Drummond A, Nicholls G, Rodrigo A, Solomon W (2002) Estimating mutation pa- rameters, population history and genealogy simultaneously from temporally spaced sequence data. Genetics 161: 1307–20. 12136032 10.1093/genetics/161.3.1307 PMC1462188

[7] Rodrigo AG, Goode M, Forsberg R, Ross HA, Drummond A (2003) Inferring evolutionary rates using serially sampled sequences from several populations. Mol Biol Evol 20: 2010–2018. 12949147 10.1093/molbev/msg215

[8] Achaz G, Palmer S, Kearney M, Maldarelli F, Mellors JW, et al (2004) A robust measure of HIV-1 population turnover within chronically infected individuals. Mol Biol Evol 21: 1902–1912. 15215321 10.1093/molbev/msh196

[9] Kingman JFC (1982) On the genealogy of large populations. Journal of Applied Probability 19A: 27–43.

[10] Kingman JFC (1982) Exchangeability and the evolution of large populations. In: Koch G, Spizzichino F, editors, Exchangeability in Probability and Statistics, North Holland Publishing Company. 97–112.

[11] Kingman JFC (1982) The coalescent. Stochastic Processes and their Applications 13: 235–248.

[12] Hudson RR (1983) Testing the constant-rate neutral allele model with protein sequence data. Evolution 37: 203–217. 28568026 10.1111/j.1558-5646.1983.tb05528.x

[13] Tajima F (1983) Evolutionary relationship of DNA sequences in finite populations. Genetics 105: 437–460. 6628982 10.1093/genetics/105.2.437 PMC1202167

[14] Hudson RR (1983) Properties of a neutral allele model with intragenic recombination. Theoretical Population Biology 23: 183–201. 6612631 10.1016/0040-5809(83)90013-8

[15] Hudson RR (1991) Gene genealogies and the coalescent process. In: Futuyma D, Antonovics J, editors, Oxford Surveys in Evolutionary Biology, Oxford University Press, volume 7: 1–44.

[16] Shriner D, Rodrigo AG, Nickle DC, Mullins JI (2004) Pervasive genomic recombi- nation of HIV-1 in vivo. Genetics 167: 1573–1583. 15342499 10.1534/genetics.103.023382 PMC1470992

[17] Kuhner MK, Yamato J, Felsenstein J (2000) Maximum likelihood estimation of recombination rates from population data. Genetics 156: 1393–1401. 11063710 10.1093/genetics/156.3.1393 PMC1461317

[18] Hudson RR (2002) Generating samples under a Wright-Fisher neutral model of genetic variation. Bioinformatics 18: 337–338. 11847089 10.1093/bioinformatics/18.2.337

[19] McVean G, Awadalla P, Fearnhead P (2002) A coalescent-based method for detecting and estimating recombination from gene sequences. Genetics 160: 1231–1241. 11901136 10.1093/genetics/160.3.1231 PMC1462015

[20] Rodrigo AG, Felsenstein J (1999) Coalescent approaches to HIV population genetics. In: Crandall K, editor, The evolution of HIV, Johns Hopkins Univ. Press, Baltimore. 233–272.

[21] Shankarappa R, Margolick JB, Gange SJ, Rodrigo AG, Upchurch D, et al (1999) Consistent Viral Evolutionary Changes Associated with the Progression of Human Immunodeficiency Virus Type 1 Infection. Journal of Virology 73: 10489–502. 10559367 10.1128/jvi.73.12.10489-10502.1999 PMC113104

[22] Tajima F (1996) The amount of DNA polymorphism maintained in a finite pop- ulation when the neutral mutation rate varies among sites. Genetics 143: 1457–65. 8807315 10.1093/genetics/143.3.1457 PMC1207412

[23] Watterson GA (1975) On the number of segregating sites in genetical models without recombination. Theoretical Population Biology 7: 256–276. 1145509 10.1016/0040-5809(75)90020-9

[24] Wakeley J (2008) An introduction to coalescent theory. Roberts & Co.

[25] Nordborg M (2001) Coalescent theory. In: D Balding MB, Cannings C, editors, Handbook of Statistical Genetics, Wiley, Chichester, UK.

[26] Pybus OG, Rambaut A, Harvey PH (2000) An integrated framework for the in- ference of viral population history from reconstructed genealogies. Genetics 155: 1429–1437. 10880500 10.1093/genetics/155.3.1429 PMC1461136

[27] Strimmer K, Pybus OG (2001) Exploring the demographic history of DNA sequences using the generalized skyline plot. Mol Biol Evol 18: 2298–2305. 11719579 10.1093/oxfordjournals.molbev.a003776

[28] Drummond A, Forsberg R, Rodrigo AG (2001) The inference of stepwise changes in substitution rates using serial sequence samples. Mol Biol Evol 18: 1365–1371. 11420374 10.1093/oxfordjournals.molbev.a003920

[29] Drummond AJ, Rambaut A, Shapiro B, Pybus OG (2005) Bayesian coalescent inference of past population dynamics from molecular sequences. Mol Biol Evol 22: 1185–1192. 15703244 10.1093/molbev/msi103

[30] Opgen-Rhein R, Fahrmeir L, Strimmer K (2005) Inference of demographic history from genealogical trees using reversible jump Markov chain Monte Carlo. BMC Evol Biol 5: 6. 15663782 10.1186/1471-2148-5-6 PMC548300

[31] Griffiths RC, Tavaré S (1994) Sampling theory for neutral alleles in a varying environment. Phil Trans R Soc Lond B 344: 403–410. 7800710 10.1098/rstb.1994.0079

[32] Robertson DL, Sharp PM, McCutchan FE, Hahn BH (1995) Recombination in HIV-1. Nature 374: 124–126. 10.1038/374124b0 7877682

[33] Schierup MH, Hein J (2000) Consequences of recombination on traditional phylo- genetic analysis. Genetics 156: 879–891. 11014833 10.1093/genetics/156.2.879 PMC1461297

[34] Anderson CN, Ramakrishnan U, Chan YL, Hadly EA (2005) Serial SimCoal: A population genetic model for data from multiple populations and points in time. Bioinformatics 21: 1733–4. 15564305 10.1093/bioinformatics/bti154

[35] Jakobsson M (2009) COMPASS: a program for generating serial samples under an infinite sites model. Bioinformatics 25: 2845–7. 19762347 10.1093/bioinformatics/btp534

[36] Excoffier L, Novembre J, Schneider S (2000) SIMCOAL: A general coalescent program for the simulation of molecular data in interconnected populations with arbitrary demography. J Heredity 91: 506–510. 10.1093/jhered/91.6.506 11218093

[37] Tajima F (1989) The effect of change in population size on DNA polymorphism. Genetics 123: 597–601. 2599369 10.1093/genetics/123.3.597 PMC1203832

[38] Batorsky R, Kearney MF, Palmer SE, Maldarelli F, Rouzine IM, et al (2011) Estimate of effective recombination rate and average selection coefficient for HIV in chronic infection. Proc Natl Acad Sci USA 108: 5661–5666. 21436045 10.1073/pnas.1102036108 PMC3078368

[39] Neher RA, Leitner T (2010) Recombination rate and selection strength in HIV intra-patient evolution. PLoS Comput Biol 6: e1000660. 20126527 10.1371/journal.pcbi.1000660 PMC2813257

[40] Weber J (2001) The pathogenesis of HIV-1 infection. Br Med Bull 58: 61–72. 11714624 10.1093/bmb/58.1.61

[41] Ariyoshi K, Harwood E, Chiengsong-Popov R, Weber J (1992) Is clearance of HIV- 1 viraemia at seroconversion mediated by neutralising antibodies? The Lancet 340: 1257–1258. 10.1016/0140-6736(92)92953-d 1359323

[42] Holmes EC, Zhang L, Simmonds P, Ludlam C (1992) Convergent and divergent sequence evolution in the surface envelope glycoprotein of human immunodefficiency virus type 1 within a single infected patient. Proc Natl Acad Sci USA 89: 4835–4839. 1594583 10.1073/pnas.89.11.4835 PMC49182

[43] Jukes TH, Cantor CR (1969) Evolution of protein molecules. In: Munro HN, editor, Mammalian Protein Metabolism, Academic Press, New York. 21–123.

[44] Kimura M (1980) A simple method for estimating evolutionary rates of base substi- tutions through comparative studies of nucleotide sequences. Journal of Molecular Evolution 16: 111–120. 7463489 10.1007/BF01731581

[45] Goldman N, Yang Z (1994) A codon-based model of nucleotide substitution for protein-coding DNA sequences. Mol Biol Evol 11: 725–36. 7968486 10.1093/oxfordjournals.molbev.a040153

[46] Yang Z (1996) Statistical properties of a DNA sample under the finite-sites model. Genetics 144: 1941–50. 8978077 10.1093/genetics/144.4.1941 PMC1207741

[47] Yang Z (1996) Among-site rate variation and its impact on phylogenetic analyses. Trends Ecol Evol 11: 367–72. 21237881 10.1016/0169-5347(96)10041-0

[48] Nielsen R, Yang Z (1998) Likelihood models for detecting positively selected amino acid sites and applications to the HIV-1 envelope gene. Genetics 148: 929–36. 9539414 10.1093/genetics/148.3.929 PMC1460041

[49] Griffiths RC, Tavaré S (1998) The age of a mutation in a general coalescent tree. Stochastic Models 14: 273–295.

[50] Griffiths R (1981) Transient distribution of the number of segregating sites in a neutral infinite-sites model with no recombination. J Appl Prob 18: 42–51.

[51] Perlitz M, Stephan W (1997) The mean and variance of the number of segregating sites since the last hitchhiking event. J Math Biol 36: 1–23. 9440302 10.1007/s002850050087

[52] Sargsyan O (2012) Analytical framework for identifying and differentiating recent hitchhiking and severe bottleneck effects from multi-locus DNA sequence data. PLoS One 7: e37588. 22662176 10.1371/journal.pone.0037588 PMC3360760

[53] Lewontin RC (1964) The interaction of selection and linkage. I. General consider- ations; heterotic models. Genetics 49: 49–67. 17248194 10.1093/genetics/49.1.49 PMC1210557

[54] Hedrick PW (1987) Gametic disequilibrium measures: proceed with caution. Ge- netics 117: 331–341. 10.1093/genetics/117.2.331 PMC1203208 3666445

[55] Tavaré S, Balding DJ, Griffiths RC, Donnelly P (1997) Inferring coalescence times from DNA sequence data. Genetics 145: 505–518. 9071603 10.1093/genetics/145.2.505 PMC1207814

[56] Anisimova M, Nielsen R, Yang Z (2003) Effect of recombination on the accuracy of the likelihood method for detecting positive selection at amino acid sites. Genetics 164: 1229–1236. 12871927 10.1093/genetics/164.3.1229 PMC1462615

[57] Bonhoeffer S, Holmes EC, Nowak MA (1995) Causes of HIV diversity. Nature 376: 125. 7603560 10.1038/376125a0

[58] Edwards CTT, Holmes EC, Pybus OG, Wilson DJ, Viscidi RP, et al (2006) Evolution of the human immunodeficiency virus envelope gene is dominated by purifying selection. Genetics 174: 1441–1453. 16951087 10.1534/genetics.105.052019 PMC1667091

