# Supplementary material for: A Framework Including Recombination for Analyzing the Dynamics of Within-Host HIV Genetic Diversity
Source: PLoS One. 2014 Feb 7;9(2):e87655. doi: 10.1371/journal.pone.0087655 (PMC3917834; doi:10.1371/journal.pone.0087655)
Supplement: Appendix S1 — The proof of Lemma 1. This section shows the derivation of the formula (2) by using the developed representation for polymorphisms in samples of DNA sequences under the piecewise constant population size model and the memoryless property of coalescence waiting times in the standard coalescent. (PDF) [file pone.0087655.s001.pdf]

Appendix: The proof of Lemma 1

Polymorphic sites in a sample of n DNA sequences taken at time point T_i are the result of the mutation events that occur on the lineages that trace the ancestral history of the sequences back in time to the latest time point before time T_0 and the most recent common ancestor of the sample. Let this part of the genealogy of the sequences denote by $G_{n,i,0}$. Under the infinite-sites model and for a given $G_{n,i,0}$, the number of polymorphic sites $S_n(T_n)$ in the n sequences is the number of mutation events on $G_{n,i,0}$. On the other hand, this number is the sum of the numbers of mutation events on the parts of $G_{n,i,0}$ between the time points T_j and T_{j-1}, $j = 1, \ldots, i$. Let these parts of the genealogy denote by $G_{n,i,0}(j)$, respectively, and let the $M(G)$ be the number of mutation events on G. Thus, the following equation holds

$$S_n(T_i) \mid G_{n,i,0} = M(G_{n,i,0}) = \sum_{j=1}^{i} M(G_{n,i,0}(j)).$$

Because of the property of the conditional expectation the expected number of polymorphic sites in the n sequences can be represented as follows

$$\mathbb{E}S_n(T_i) = \mathbb{E}\mathbb{E}(S_n(T_i) \mid G_{n,i,0}) = \sum_{j=1}^{i} \mathbb{E}\mathbb{E}M(G_{n,i,0}(j)).$$

The right side of the above equation can be modified further based on the fact that mutation events on the branches of $G_{n,i,0}(j)$ occur as a Poisson process with rate $\theta_j/2$. Therefore, the following equation holds

$$\mathbb{E}\mathbb{E}M(G_{n,i,0}(j)) = \frac{\theta_j}{2}\mathbb{E}L(G_{n,i,0}(j)),$$

in which $L(G_{n,i,0}(j))$ is the total branch length of $G_{n,i,0}(j)$. The expectation $\mathbb{E}L(G_{n,i,0}(j))$ can be modified further by conditioning on the number of the lineages at time T_j. Let $d_{n,i,0}(j)$ be the number of the lineages in $G_{n,i,0}$ at time T_j. Thus, the following equation holds

$$\mathbb{E}L(G_{n,i,0}(j)) = \mathbb{E}\mathbb{E}(L(G_{n,i,0}(j)) \mid d_{n,i,0}(j)).$$

Since the waiting times between consecutive coalescent events in $G_{n,i,0}$ are exponential random variables which satisfy the memoryless property, under the condition $d_{n,i,0}(j) = d$, in which $d = 2, \ldots, n$, the following equation holds

$$G_{n,i,0}(j) \mid d_{n,i,0}(j) = G_{d,j,j-1},$$

$G_{d,j,j-1}$ is represents the lineages of d sequences at time point T_j that trace the ancestral history of the d sequences back in time to the latest time point before time T_{j-1} and

the most recent common ancestor of the sample. After combining this equation with the above equations gives the following equation

$$\mathbb{E}S_n(T_i) = \sum_{j=1}^{i} \sum_{d=2}^{n} \frac{\theta_j}{2} \mathbb{E}L(G_{d,j,j-1}) \mathbb{P}(d_{n,i,0}(j) = d),$$

in which $\mathbb{E}L(G_{d,j,j-1})$ is equal to $\phi_d(\tau_j)$ and $\mathbb{P}(d_{n,i,0}(j) = d)$ is equal to $\mathbb{P}\left(n, d, \sum_{k=j+1}^{i} \tau_k\right)$. Thus, the proof of the lemma is complete.
